# Sex difference in the association between plasma selenium and first stroke: a community-based nested case-control study

**DOI:** 10.1186/s13293-021-00383-2

**Published:** 2021-05-29

**Authors:** Huan Hu, Chonglei Bi, Tengfei Lin, Lishun Liu, Yun Song, Binyan Wang, Ping Wang, Ziyi Zhou, Chongqian Fang, Hai Ma, Xiao Huang, Lihua Hu, Xiping Xu, Hao Zhang, Yong Huo, Xiaobin Wang, Huihui Bao, Xiaoshu Cheng, Ping Li

**Affiliations:** 1 Department of Cardiovascular Medicine, The Second Affiliated Hospital of Nanchang University, No. 1 Minde Road, Nanchang, Jiangxi Province China; 2 Center for Prevention and Treatment of Cardiovascular Diseases, The Second Affiliated Hospital of Nanchang University, Nanchang, No. 1 Minde Road, Nanchang, Jiangxi Province China; 3 People’s Hospital of Rongcheng, No. 298 Chengshan Avenue, Rongcheng, Shandong Province China; 4 Beijing Advanced Innovation Center for Food Nutrition and Human Health, College of Food Science and Nutritional Engineering, China Agricultural University, No. 17 Tsinghua East Road, Beijing, China; 5 Institute of Biomedicine, Anhui Medical University, No. 81 Meishan Road, Hefei, Anhui Province China; 6 Shenzhen Evergreen Medical Institute, No. 16 Gaoxin Middle 1 Road, Shenzhen, China; 7 School of Public Health (Shenzhen), Sun Yat-Sen University, No. 135 Xingang West Road, Guangzhou, Guangdong Province China; 8 Health and Family Planning Commission, No. 688 Qingshan East Road, Rongcheng, Shandong Province China; 9 Department of Cardiology, Peking University First Hospital, No. 8 Xishiku Street, Beijing, China; 10 Department of Population, Family and Reproductive Health, Johns Hopkins University Bloomberg School of Public Health, 3400 N. Charles Street, Baltimore, MD 21205 USA

**Keywords:** Selenium, First stroke, First ischemic stroke, First hemorrhagic stroke, Vitamin E

## Abstract

**Background:**

To date, there is no clearly defined association between plasma selenium levels and first stroke. We aimed to investigate the association between baseline plasma selenium and first stroke risk in a community-based Chinese population.

**Methods:**

Using a nested case-control study design, a total of 1255 first stroke cases and 1255 matched controls were analyzed. Participant plasma selenium concentrations were measured by inductively coupled plasma mass spectrometry (ICP-MS), and the association of plasma selenium with first stroke risk was estimated by conditional logistic regression models.

**Results:**

Overall, a non-linear negative association between plasma selenium and first total stroke and first ischemic stroke risks was found in males but not in females. Compared with participants with lower selenium levels (tertile 1–2, < 94.1 ng/mL), participants with higher selenium levels (tertile 3, ≥ 94.1 ng/mL) had significantly lower risks of first total stroke (OR 0.63; 95% CI 0.48, 0.83) and first ischemic stroke (OR 0.61; 95% CI 0.45, 0.83) in males but not in females with first total stroke (OR 0.92; 95% CI 0.69, 1.22) and first ischemic stroke (OR 0.89; 95% CI 0.65, 1.22). Furthermore, a stronger association between plasma selenium and first total stroke was found in males with higher vitamin E levels (≥ 13.5 μg/mL *vs.* < 13.5 μg/mL *P*-interaction = 0.007). No significant association was observed between plasma selenium and first hemorrhagic stroke risk in either males or females.

**Conclusion:**

Our study indicated a significant, non-linear, negative association between plasma selenium and first stroke in males but not in females.

**Trial registration:**

ChiCTR1800017274.

**Supplementary Information:**

The online version contains supplementary material available at 10.1186/s13293-021-00383-2.

## Highlights

This study is the first work to find a significant non-linear, inverse association between baseline plasma selenium and first total stroke and first ischemic stroke risks in males but not in females. The present study is also the first to indicate a stronger non-linear negative relationship between plasma selenium and first stroke in male participants with higher plasma vitamin E levels (≥ 13.5 μg/mL) than in those with lower plasma vitamin E levels (< 13.5 μg/mL). This study found no significant association between plasma selenium and first hemorrhagic stroke risk in either males or females.

## Introduction

Stroke is a leading cause of mortality and disability worldwide. Accounting for almost one-third of stroke mortality worldwide, China bears the heaviest stroke burden in the world [1, 2]. Since control of risk factors for stroke helps decrease stroke burden [3, 4], the identification of novel risk factors is urgent to further lower stroke risk. The potential effects of nutritional determinants on stroke have attracted increasing attention for controlling the occurrence of stroke. Appropriate intake of electrolyte minerals, one type of micronutrient, plays a vital role in maintaining brain health. There was an inverse association between potassium intake and stroke risk, whereas higher sodium intake was associated with a higher risk of stroke [5, 6]. We previously found that there was a significant inverse association between plasma zinc and first hemorrhagic stroke [7], whereas excess zinc also led to neurotoxicity [8]. Thus, appropriate intake of nutritional determinants and maintaining their reasonable levels play important roles in controlling stroke risk. Although accumulating evidence has indicated that trace elements might exert effects on stroke [9, 10], there is still limited evidence of dietary supplementation for stroke prevention [11], suggesting that much more evidence is needed to clarify this issue.

Selenium (Se), an essential trace element, acts as the active center of selenoproteins or selenoenzymes (e.g., glutathione peroxidases), which have many important biological functions, including antioxidant, anti-inflammatory, and immunoregulatory roles [12–14]. Insufficient or excessive Se intake may be associated with many adverse health outcomes [15–17]. Se overexposure might have toxic effects, which should not be ignored, as high Se levels have been reported to be positively associated with type 2 diabetes, and this association might exists only in females [18–21], whereas Se deficiency is mainly positively associated with Keshan disease and Kashin-Beck disease [22, 23]. Low Se status was also reported to be related to an increased risk of thyroid disease, including autoimmune thyroiditis, subclinical hypothyroidism, and hypothyroidism, and Se deficiency might constitute a risk factor for hyperthyroidism, especially in males [24, 25]. Low serum Se was an independent predictor of cancer mortality only in male participants with a median plasma Se concentration of 75.9 ng/mL, while another nested case-control study with a mean plasma Se concentration of 84.0 ng/mL from the European Prospective Investigation into Cancer and Nutrition indicated that Se status is suboptimal in many Europeans (with low Se status) and suggested an inverse association between colorectal cancer risk and higher serum Se status, which was more evident in females [26, 27]. Previous studies from two prominent Se intervention trials in North America (with currently high selenium status) observed conflicting results with regard to Se intake and cancer risk, which indicated that an interaction of Se status and cancer risk could be found in the Nutritional Prevention of Cancer Trial (NPC) [28] with a median plasma Se concentration of 115 ng/mL at baseline but not in Selenium and Vitamin E Cancer Prevention Trial (SELECT) [29] with a median plasma Se concentration of 136 ng/mL at baseline. High Se status was also positively associated with cognitive performance in males only among the population of the Nation Health and Nutrition Survey (NHANES, 2011–2014), with a median whole blood Se concentration of 196.7 ng/mL [30]. Thus, there was an interaction of Se with diabetes, thyroid disease, cancers, and cognitive performance, which seemed to be associated with sex differences and needs to be further understood. Different baseline Se levels and any other poorly defined confounders might affect these complex associations. In particular, health problems caused by Se deficiency need to be given great attention in China, a Se-deficient country where it is estimated that over 105 million people potentially face adverse health impacts due to Se deficiency [31, 32]. Based on the aforementioned conclusions, more studies are still needed to identify the appropriate level of Se that should be considered safe to address the uncertainties regarding the health risks of Se exposure. Although cross-sectional epidemiologic studies have indicated inverse associations between Se levels and stroke risk [33–35], previous prospective epidemiologic studies have reported inconsistent findings on the associations between Se concentrations and stroke risk [36–38]. Moreover, few studies have thoroughly analyzed the potential modifiers affecting this association; in particular, whether this relationship was associated with sex differences remains unclear. Therefore, the prospective relationship between plasma Se and the risk of first stroke remains inconclusive and deserves further investigation.

To narrow the knowledge gap mentioned above, we performed a nested case-control study to investigate the association between baseline plasma Se levels and the risk of first total stroke and stroke subtypes (ischemic stroke and hemorrhagic strokes) and examined any possible effect modifiers using data from a community-based population in China.

## Methods

### Study population and design

Our present study is a subset of the China H-type Hypertension Registry Study (CHHRS; URL: http://www.chictr.org.cn; Unique identifier: ChiCTR1800017274), which is an ongoing community-based non-intervention, prospective, observational, multicenter, real-world registry study and was mainly conducted in Rongcheng County, Shandong Province, and Lianyungang, Jiangsu Province, China. It was designed to establish a national registry of patients with hypertension, to investigate the prevalence and treatment of H-type hypertension in China and the related factors affecting its prognosis, and finally to construct a risk prediction model of cardio-cerebral and renal vascular diseases. Eligible participants were men and women aged ≥ 18 years with essential hypertension, defined as seated systolic blood pressure (SBP) ≥ 140 mmHg and/or seated diastolic blood pressure (DBP) ≥ 90 mmHg at the screening visit. The boundaries for elevated blood pressure (140 and 90 mmHg, respectively) were applied irrespective of age. Participants were excluded if they had psychological or nervous system impairment resulting in an inability to demonstrate informed consent or were unable to be followed-up according to the study protocol. The trial consisted of two stages: screening and recruitment and a 3-year observation follow-up period. Participants were scheduled for follow-up every 3 months. At each visit, blood pressure, heart rate, the usage of medications, adverse events, and study outcome events were measured and recorded. The primary outcome was the first composite of cardiovascular events consisting of non-fatal stroke, myocardial infarction, vascular death, and all-cause death.

The current nested case-control study utilized data from the CHHRS, which was conducted in Rongcheng, a coastal area of Shandong Province, China. This study matched stroke cases with an equal number of controls (patients without stroke) by age ± 1 year, sex, and village. Patients with stroke data from the Chinese Centers for Disease Control and Prevention (CDC, 2016-2018) who had complete records (physical exam, questionnaire, and biological samples) were selected as cases. The initial sample consisted of 1401 incident cases and 1401 matched controls, both of which were recruited from the same trial population. Next, we excluded participants with missing serum selenium values (n = 287) and unpaired individuals (n = 5). Based on the inclusion and exclusion criteria, 1255 stroke cases and 1255 matched controls with complete Se measurements were selected for final data analysis (Supplementary Fig. 1).

### Ethics

The present study was approved by the Ethics Committee of the Institute of Biomedicine, Anhui Medical University, Hefei, China. All participants signed an approved written consent form after the study protocol was thoroughly explained to them.

### Outcomes

The primary outcome of the present study was a first non-fatal or fatal stroke. Secondary outcomes included first ischemic stroke (fatal and non-fatal) and first hemorrhagic stroke (fatal and non-fatal).

Information on the incidence of first stroke for all participants was obtained via the Centers for Disease Control and Prevention of Rongcheng County and checked against the national health insurance system with electronic linkage to all hospitalizations or ascertained through active follow-up. Diseases were coded according to the International Classification of Diseases, 10th Revision (ICD-10). Secondary outcomes included first ischemic stroke (I63) and first hemorrhagic stroke (I60-I61). The primary outcome (first non-fatal or fatal stroke) included first ischemic stroke (I63), first hemorrhagic stroke (I60-I61), and no type stroke (I64).

According to government regulations, local authorities from medical institutions are required to report all new cases of stroke to the local Centers for Disease Control and Prevention. A report card that includes information on demographics, diagnostic basis and date of stroke is required to be submitted on the 28th of each month. Quality control, including finding and deleting repeated cases, error checking, and determining any missed cases, is completed by trained officials. Furthermore, the staff from the local Centers for Disease Control and Prevention double checked these information and were responsible for deleting repeated cases and finding logistical errors and missed cases. In addition, 5% of all uploaded cases were randomly chosen for further confirmation by phone or door-to-door interviews.

### Laboratory assays

Baseline serum total homocysteine (tHcy), fasting glucose levels, and lipids were measured using automatic clinical analyzers (Beckman Coulter, AU680) at the Shenzhen Tailored Medical Laboratory in Shenzhen, China. Estimated glomerular filtration rates (eGFRs) were estimated by the Chronic Kidney Disease Epidemiology Collaboration equation. Baseline plasma vitamin E concentrations were measured using liquid chromatography–tandem quadrupole mass spectrometry (LC-MS/MS), and plasma selenium (Se) concentrations were measured by inductively coupled plasma mass spectrometry (ICP-MS) using a Thermo Fisher iCAP Q ICP-MS in a commercial laboratory (Beijing DIAN Medical Laboratory, China). In the present study, the intra-assay CV for Se ranged from 1.02 to 7.93%, while the inter-assay CV for Se ranged from 2.79 to 3.51%. According to a previous study [39], the reference value (50–120 ng/mL) for plasma Se levels was used in this study.

### Statistical analysis

Baseline characteristics are presented as the means ± SDs for continuous variables and as frequencies (%) for categorical variables. Differences in baseline characteristics between males and females and cases and controls were compared using chi-square tests for categorical variables and *t* tests for continuous variables. Differences in population characteristics according to Se tertiles were compared using ANOVA tests, or chi-square tests.

Variables that are known as traditional or suspected risk factors for stroke [40] and matched variables or variables that showed significant differences between cases and controls were adjusted for in the models. Odds ratios (ORs) and 95% confidence intervals (95% CIs) for first stroke, first ischemic stroke, and first hemorrhagic stroke were calculated by modeling plasma Se as tertiles using conditional logistic regression, without and with adjustment for matched variables (sex and age), body mass index (BMI), baseline systolic blood pressure (SBP), baseline diastolic blood pressure (DBP), smoking status, alcohol consumption, labor intensity, baseline total homocysteine (tHcy), plasma vitamin E, fasting glucose, estimated glomerular filtration rate (eGFR), anti-platelet drugs, lipoprotein-lowering drugs, glucose-lowering drugs, anti-hypertensive drugs, self-reported hypertension, self-reported diabetes, self-reported atrial fibrillation, and self-reported hyperlipidemia. A generalized additive model (GAM) and smooth curve fitting (penalized spline method) were evaluated to further characterize the shape of the association between serum Se and first stroke and its subtypes. As additional exploratory analyses, possible modifications of the association between plasma Se (tertile 3, ≥ 94.1 *vs.* tertile 1–2, < 94.1 ng/mL) and first total stroke in male and female participants were also assessed for variables including age (< 70 *vs.* ≥ 70 years), BMI (< 24 *vs.* ≥ 24 kg/m^2^), current smoking (yes *vs.* no), current alcohol consumption (yes *vs.* no), baseline SBP (< 140 *vs.* ≥ 140 mmHg), fasting glucose (< 6.1 *vs.* ≥ 6.1 mmol/L or diabetes), total cholesterol (< 5.8 [median] *vs.* ≥ 5.8 mmol/L), triglycerides (< 1.2 [median] *vs.* ≥ 1.2 mmol/L), estimated glomerular filtration rate (< 90 *vs.* ≥ 90 mL/min/1.73 m^2^), total homocysteine (< 12.5 [median] *vs.* ≥ 12.5 μmol/L), and vitamin E (< 13.5 [median] *vs.* ≥ 13.5 μmol/L) using multivariate logistic regression models. Diabetes was defined as fasting serum glucose ≥ 7.0 mmol/L, self-reported use of anti-diabetic medications, or physician-diagnosed diabetes. Potential interactions were examined by including the interaction terms in those logistic regression models with the greatest number of confounding variables.

A 2-tailed *P* < 0.05 was considered to be statistically significant in all analyses. R software version 3.4.3 (www.R-project.org) and Empower version 2.17.8 (www.empowerstats.com, X&Y Solutions, Inc.) were used for all statistical analyses.

## Results

### Study participants and baseline characteristics

A total of 1255 first stroke cases (1079 cases of first ischemic stroke, 171 cases of first hemorrhagic stroke, and 5 cases of first uncertain type of stroke) and 1255 matched controls were included in this analysis. The mean age of all participants at baseline was 70.8 years (SD, 8.1), 49.5% of the participants were male, and the mean Se level was 87.2 ng/mL (SD, 18.6). The baseline characteristics of the male and female participants are shown in Table 1. The reference value of 50–120 ng/mL for serum Se concentration was used in this study. The detailed plasma Se concentration distribution of subjects is listed in Supplemental Table 1, and 95.1% of the participants were within the normal range of Se levels with respect to the reference value (50–120 ng/mL) for plasma Se concentrations. As shown in Table 1, male participants had non-significantly higher Se levels (87.7 ± 18.6 *vs.* 86.8 ± 18.6 ng/mL; *P* = 0.230) and significantly lower vitamin E (12.9 ± 3.3 *vs.* 15.2 ± 4.3 ng/mL; *P* < 0.001) than females. The distribution graph of serum Se and vitamin E levels in males versus females showed similar results (Supplemental Fig. 2A-B). Males also tended to be older, were more likely to be current smokers and current drinkers, had higher DBP and tHcy levels, as well as lower BMI, SBP, TC, TG, and glucose levels at baseline and a lower frequency of lipid-lowering, glucose-lowering, and anti-hypertensive drug use, and were less likely to be hypertensive patients or self-reported diabetic and self-reported hyperlipidemia patients compared with female participants.

**Table 1:** Baseline characteristics of male and female participants^a^

Characteristics	Total	Male	Female	*P* value
N	n = 2510	n=1242	n = 1268	
Age, years	70.8 ± 8.1	71.4 ± 8.1	70.1 ± 8.0	< 0.001
BMI, kg/m^2^	26.2 ± 4.1	25.3 ± 3.6	27.0 ± 4.4	< 0.001
Current smoking, n (%)	551 (22.0)	547 (44.0)	4 (0.3)	< 0.001
Current alcohol drinking, n (%)	612 (24.4)	599 (48.2)	13 (1.0)	< 0.001
Baseline SBP, mmHg	153.3 ± 23.1	150.8 ± 22.3	155.7 ± 23.6	< 0.001
Baseline DBP, mmHg	85.3 ± 12.3	86.2 ± 12.5	84.3 ± 12.0	< 0.001
Self-reported hypertension, n (%)	1312 (52.3)	553 (44.5)	759 (59.9)	< 0.001
Self-reported diabetes, n (%)	424 (16.9)	161 (13.0)	263 (20.7)	< 0.001
Self-reported hyperlipidemia, n (%)	264 (10.5)	108 (8.7)	156 (12.3)	0.003
Self-reported atrial fibrillation, n (%)	46 (1.8)	26 (2.1)	20 (1.6)	0.335
Hypertension, n (%)^b^	2016 (80.3)	940 (75.7)	1076 (84.9)	< 0.001
Labor intensity, n (%)				< 0.001
Mild	1885 (75.1)	887 (71.4)	998 (78.7)	
Moderate	488 (19.4)	280 (22.5)	208 (16.4)	
Severe	137 (5.5)	75 (6.0)	62 (4.9)	
**Medication use, n (%)**				
Anti-platelet drugs	83 (3.3)	47 (3.8)	36 (2.8)	0.186
Lipid-lowering drugs	44 (1.8)	15 (1.2)	29 (2.3)	0.039
Glucose-lowering drugs	301 (12.0)	112 (9.0)	189 (14.9)	< 0.001
Anti-hypertensive drugs	1152 (45.9)	476 (38.3)	676 (53.3)	< 0.001
**Laboratory results**				
TC, mmol/L	5.9 ± 1.2	5.6 ± 1.1	6.1 ± 1.3	< 0.001
TG, mmol/L	1.4 ± 0.9	1.2 ± 0.7	1.6 ± 0.9	< 0.001
HDL-C, mmol/L	1.6 ± 0.4	1.6 ± 0.4	1.6 ± 0.4	0.558
Glucose, mmol/L	6.3 ± 2.3	6.1 ± 2.1	6.4 ± 2.5	< 0.001
tHcy, μmol/L	13.9 ± 7.2	15.3 ± 8.6	12.5 ± 5.0	< 0.001
eGFR, mL/min/1.73 m^2^	92.5 ± 14.4	92.3 ± 15.1	92.7 ± 13.8	0.428
Vitamin E, μg/mL	14.0 ± 4.0	12.9 ± 3.3	15.2 ± 4.3	< 0.001
Selenium, ng/mL	87.2 ± 18.6	87.7 ± 18.6	86.8 ± 18.6	0.230

*Abbreviations*: *BMI* body mass index, *SBP* systolic blood pressure, *DBP* diastolic blood pressure, *TC* total cholesterol, *TG* triglycerides, *HDL-C* high-density lipoprotein-cholesterol, *tHcy* total homocysteine, *eGFR* estimated glomerular filtration rate

^a^Variables are presented as the mean ± SD or n (%)

^b^Hypertension was defined as a self-reported history of hypertension, use of anti-hypertensive drugs, SBP ≥ 140 mmHg, or DBP ≥ 90 mmHg

The baseline characteristics of cases and control participants by sex are shown in Table 2. For stroke cases, male stroke cases had non-significantly lower Se levels than females (86.2 ± 16.8 *vs.* 87.0 ± 18.4 ng/mL; *P* = 0.436). Male stroke cases also tended to be older, were more likely to be current smokers and current drinkers, had higher DBP and tHcy levels, as well as lower BMI, SBP, TC, TG, glucose, and vitamin E levels at baseline and a lower frequency of glucose-lowering and anti-hypertensive drug use, and were less likely to be hypertensive patients or self-reported diabetic and self-reported hyperlipidemia patients compared with female stroke cases. For non-stroke controls, male non-stroke controls had significantly higher Se levels than females (89.1 ± 20.1 *vs.* 86.6 ± 18.7 ng/mL; *P* = 0.020). Male non-stroke controls also tended to be older, were more likely to be current smokers, current drinkers and self-reported atrial fibrillation patients, had higher DBP and tHcy levels, as well as lower BMI, SBP, TC, TG, and vitamin E levels at baseline and a lower frequency of glucose-lowering and anti-hypertensive drug use, and were less likely to be hypertensive patients or self-reported diabetic patients compared with female non-stroke controls.

**Table 2:** Baseline characteristics of cases and control participants by sex^a^

Characteristics	First stroke cases		*P* value	Non-stroke controls		*P* value
	Males	Females		Males	Females	
N	n = 621	n = 634		n = 621	n = 634	
Age, years	71.4 ± 8.1	70.1± 8.0	0.004	71.4 ± 8.1	70.1 ± 8.0	0.003
BMI, kg/m^2^	25.7 ± 3.6	27.3 ± 4.9	< 0.001	25.0 ± 3.5	26.7 ± 3.8	< 0.001
Current smoking, n (%)	293 (47.2)	3 (0.5)	< 0.001	254 (40.9)	1 (0.2)	< 0.001
Current alcohol drinking, n (%)	291 (46.9)	4 (0.6)	< 0.001	308 (49.6)	9 (1.4)	< 0.001
Baseline SBP, mmHg	154.8 ± 23.1	159.5 ± 24.2	< 0.001	146.8 ± 20.8	151.8 ± 22.2	< 0.001
Baseline DBP, mmHg	88.0 ± 13.4	86.4 ± 12.2	0.030	84.4 ± 11.3	82.2 ± 11.4	< 0.001
Self-reported hypertension, n (%)	319 (51.4)	427 (67.4)	< 0.001	234 (37.7)	332 (52.4)	< 0.001
Self-reported diabetes, n (%)	97 (15.6)	164 (25.9)	< 0.001	64 (10.3)	99 (15.6)	0.005
Self-reported hyperlipidemia, n (%)	46 (7.4)	83 (13.1)	< 0.001	62 (10.0)	73 (11.5)	0.382
Self-reported atrial fibrillation, n (%)	13 (2.1)	16 (2.5)	0.612	13 (2.1)	4 (0.6)	0.025
Hypertension, n (%)^b^	511 (82.3)	564 (89.0)	< 0.001	429 (69.1)	512 (80.8)	< 0.001
Labor intensity, n (%)			< 0.001			0.006
Mild	457 (73.6)	521 (82.2)		430 (69.2)	477 (75.2)	
Moderate	125 (20.1)	96 (15.1)		155 (25.0)	112 (17.7)	
Severe	39 (6.3)	17 (2.7)		36 (5.8)	45 (7.1)	
**Medication use, n (%)**						
Anti-platelet drugs	37 (6.0)	23 (3.6)	0.053	10 (1.6)	13 (2.1)	0.561
Lipid-lowering drugs	8 (1.3)	14 (2.2)	0.214	7 (1.1)	15 (2.4)	0.095
Glucose-lowering drugs	72 (11.6)	121 (19.1)	< 0.001	40 (6.4)	68 (10.7)	0.007
Anti-hypertensive drugs	281 (45.2)	383 (60.4)	< 0.001	195 (31.4)	293 (46.2)	< 0.001
**Laboratory results**						
TC, mmol/L	5.6 ± 1.1	6.1 ± 1.3	< 0.001	5.7 ± 1.1	6.1 ± 1.3	< 0.001
TG, mmol/L	1.3 ± 0.8	1.7 ± 1.0	< 0.001	1.1 ± 0.7	1.5 ± 0.8	< 0.001
HDL-C, mmol/L	1.6 ± 0.4	1.6 ± 0.4	0.935	1.7 ± 0.4	1.7 ± 0.4	0.376
Glucose, mmol/L	6.3 ± 2.2	6.8 ± 2.8	0.001	5.9 ± 1.9	6.1 ± 2.2	0.051
tHcy, μmol/L	15.8 ±9.5	12.8 ± 5.7	< 0.001	14.7 ± 7.5	12.2 ± 4.3	< 0.001
eGFR, mL/min/1.73 m^2^	91.4 ± 15.9	92.0 ± 14.7	0.485	93.1 ± 14.1	93.4 ± 12.7	0.685
Vitamin E, μg/mL	13.0 ± 3.4	15.2 ± 4.3	< 0.001	12.8 ± 3.1	15.1 ± 4.3	< 0.001
Selenium, ng/mL	86.2 ± 16.8	87.0 ± 18.4	0.436	89.1 ± 20.1	86.6 ± 18.7	0.020

*Abbreviations*: *BMI* body mass index, *SBP* systolic blood pressure, *DBP* diastolic blood pressure, *TC* total cholesterol, *TG* triglycerides, *HDL-C* high-density lipoprotein-cholesterol, *tHcy* total homocysteine, *eGFR* estimated glomerular filtration rate

^a^Variables are presented as the mean ± SD or n (%)

^b^Hypertension was defined as a self-reported history of hypertension, use of anti-hypertensive drugs, SBP ≥ 140 mmHg, or DBP ≥ 90 mmHg

In addition, plasma Se was positively associated with BMI, current smoking, current alcohol consumption, self-reported diabetes, a higher frequency of glucose-lowering drug use, TC, high-density lipoprotein cholesterol, fasting glucose, and vitamin E levels and was inversely associated with labor intensity and tHcy levels at baseline (Supplemental Table 2).

### Association between plasma selenium concentration and first stroke in all participants

**Fig. 1:**
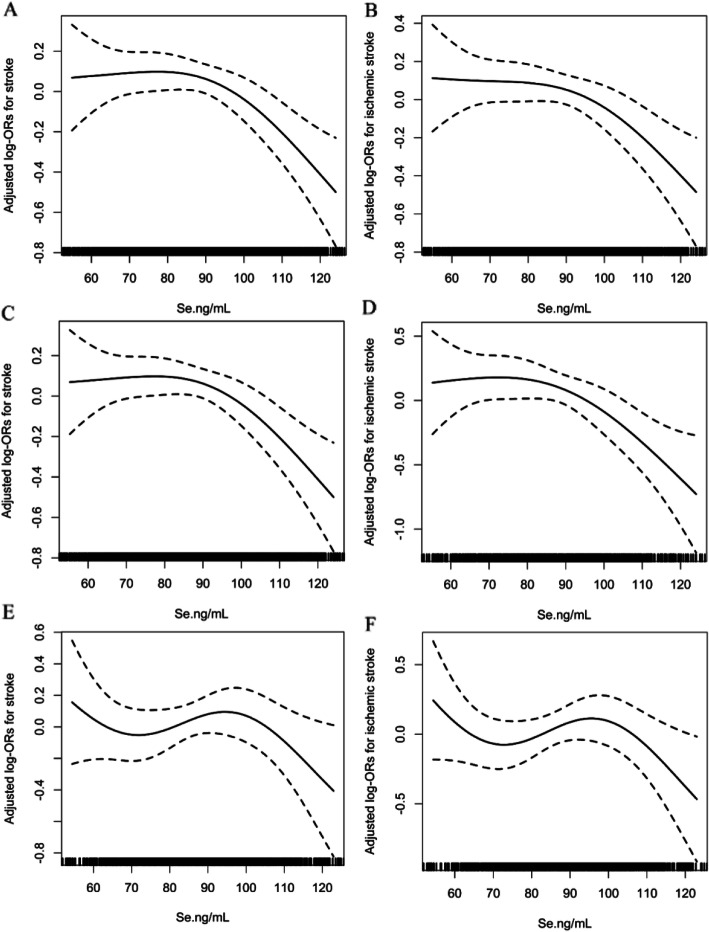
The association between baseline plasma selenium and first total stroke and first ischemic stroke risks. Odds ratios for first total stroke in the (**A**) total population, (**C**) males, and (**E**) females, and for first ischemic stroke in the (**B**) total population, (**D**) males, and (**F**) females by plasma selenium levels. In addition to the matching factors (age and sex), the splines also adjusted for BMI, baseline SBP, baseline DBP, smoking status, alcohol consumption, labor intensity, baseline total homocysteine, vitamin E, fasting glucose, estimated glomerular filtration rate (eGFR), anti-platelet drugs, lipoprotein-lowering drugs, glucose-lowering drugs, anti-hypertensive drugs, self-reported hypertension, self-reported diabetes, self-reported atrial fibrillation, and self-reported hyperlipidemia

Overall, there was a non-linear negative association between plasma Se levels and the risk of first total stroke and first ischemic stroke (Fig. 1A, B) but not with the risk of first hemorrhagic stroke (Supplemental Fig. 3A) in all participants. Consistently, when plasma Se was assessed as tertiles that were calculated among the whole population, significantly lower risks of first total stroke (Model 2, OR 0.77; 95% CI 0.61, 0.97) and first ischemic stroke (model 2, OR 0.78; 95% CI 0.60, 0.99) were found in participants in tertile 3 (≥ 94.1 ng/mL) than in those in tertile 1 (< 79.1 ng/mL) (Table 3). It is worth noting that non-significantly higher risks of first total stroke (model 2, OR 1.02; 95% CI 0.82, 1.26) and first ischemic stroke (model 2, OR 1.05; 95% CI 0.83, 1.33) were found in participants in tertile 2 (79.1 to < 94.1 ng/mL) than in those in tertile 1 (< 79.1 ng/mL) (Table 3). Due to the similar first total stroke and first ischemic stroke prevalence in participants with Se levels in tertile 1 and tertile 2 (Table 3), we combined these two groups into one group called tertile 1–2. Compared with participants with lower Se levels in tertile 1–2 (< 94.1 ng/mL), significantly lower risks of first total stroke (model 2, OR 0.76; 95% CI 0.63, 0.93) and first ischemic stroke (model 2, OR 0.75; 95% CI 0.61, 0.93) were found in those with higher Se levels in tertile 3 (≥ 94.1 ng/mL) (Table 3). However, no significant association was found between plasma Se concentrations and first hemorrhagic stroke (Table 3).

**Table 3:** Risk of first stroke (total and subtypes) associated with plasma selenium concentrations in all participants^a^

Selenium, ng/mL	Cases/controls	Model 1		Model 2	
		OR (95% CI)	*P* value	OR (95% CI)	*P* value
**First total stroke**					
*Tertiles*					
T1 (< 79.1)	420/417	*Ref.*		*Ref.*	
T2 (79.1 to < 94.1)	434/402	1.07 (0.88, 1.30)	0.516	1.02 (0.82, 1.26)	0.891
T3 (≥ 94.1)	401/436	0.90 (0.73, 1.11)	0.317	0.77 (0.61, 0.97)	0.027
*Categories*					
T1–T2 (< 94.1)	854/819	Ref.		Ref.	
T3 (≥ 94.1)	401/436	0.87 (0.73, 1.04)	0.119	0.76 (0.63, 0.93)	0.007
**Ischemic stroke**					
*Tertiles*					
T1 (< 79.1)	360/363	*Ref.*		*Ref.*	
T2 (79.1 to < 94.1)	372/340	1.10 (0.89, 1.35)	0.376	1.05 (0.83, 1.33)	0.672
T3 (≥ 94.1)	347/376	0.92 (0.74, 1.14)	0.444	0.78 (0.60, 0.99)	0.045
*Categories*					
T1–T2 (< 94.1)	732/703	*Ref.*		*Ref.*	
T3 (≥ 94.1)	347/376	0.87 (0.72, 1.06)	0.163	0.75 (0.61, 0.93)	0.009
**Hemorrhagic stroke**					
*Tertiles*					
T1 (< 79.1)	58/54	*Ref.*		*Ref.*	
T2 (79.1 to < 94.1)	60/61	0.88 (0.50, 1.57)	0.673	0.76 (0.38, 1.55)	0.455
T3 (≥ 94.1)	53/56	0.85 (0.47, 1.54)	0.587	0.72 (0.35, 1.48)	0.371
*Categories*					
T1–T2 (< 94.1)	118/115	*Ref.*		*Ref.*	
T3 (≥ 94.1)	53/56	0.92 (0.57, 1.47)	0.718	0.85 (0.48, 1.50)	0.577

^a^ ORs of first stroke (total), first ischemic, and hemorrhagic stroke in relation to plasma selenium (tertiles) were analyzed using conditional logistic regression models. Model 1 is conditioned on the matching factors of age and sex; model 2 is conditioned on the matching factors of age and sex, as well as adjusted for BMI, baseline SBP, baseline DBP, smoking status, alcohol consumption, labour intensity, baseline total homocysteine, vitamin E, fasting glucose, estimated glomerular filtration rate (eGFR), anti-platelet drugs, lipoprotein-lowering drugs, glucose-lowering drugs, anti-hypertensive drugs, self-reported hypertension, self-reported atrial fibrillation, self-reported diabetes, and self-reported hyperlipidemia.

### Association between plasma selenium concentrations and first stroke by sex

Given the differences in plasma Se levels between male and female participants (87.7 ± 18.6 *vs.* 86.8 ± 18.6 ng/mL), we further investigated the possible effect of sex on the Se-first stroke association. Overall, there was a non-linear negative association between plasma Se levels and the risks of first total stroke and first ischemic stroke in males (Fig. 1C, D) but not in females (Fig. 1E, F). Furthermore, there was no significant association between plasma Se and first hemorrhagic stroke in either sex (Supplemental Fig. 3B-C). Consistently, when the plasma Se of males and females was assessed as tertiles according to the tertile cut-off value of the whole population, the highest tertile (T3, ≥ 94.1 ng/mL) of plasma Se was associated with a lower first total stroke risk in males (model 2, OR 0.67; 95% CI 0.48, 0.93, *P =* 0.017) but not in females (model 2, OR 0.85; 95% CI 0.61, 1.19, *P =* 0.353) compared with the lowest tertile (T1, < 79.1 ng/mL) of plasma Se (Table 4). It is also worth noting that non-significantly higher risks of first total stroke (model 2, OR 1.11; 95% CI 0.80, 1.54) and first ischemic stroke (model 2, OR 1.17; 95% CI 0.82, 1.66) were found in male participants in tertile 2 (79.1 to < 94.1 ng/mL) than in those in tertile 1 (< 79.1 ng/mL) (Table 3). Accordingly, higher Se levels in tertile 3 (≥ 94.1 ng/mL) were associated with a lower first total stroke risk in males (model 2 OR 0.63; 95% CI 0.48, 0.83, *P =* 0.001), but not in females (model 2, OR 0.92; 95% CI 0.69, 1.22, *P =* 0.563) compared with lower Se levels in tertile 1–2 (< 94.1 ng/mL) (Table 4).

**Table 4:** The association of plasma selenium with the risk of first stroke (total and subtypes) by sex^a^

	^b^Male participants			Female participants		
	Cases/controls	Model 1	Model 2	Cases/controls	Model 1	Model 2
		OR (95% CI) *P* value	OR (95% CI) *P* value		OR (95% CI) *P* value	OR (95% CI) *P* value
**First total stroke**						
*Selenium tertiles*						
T1 (< 79.1 ng/mL)	206/193	*Ref.*	*Ref.*	214/224	*Ref.*	*Ref.*
T2 (79.1 to < 94.1)	218/183	1.12 (0.84, 1.49) 0.452	1.11 (0.80, 1.54) 0.519	216/219	1.04 (0.80, 1.36) 0.764	0.88 (0.65, 1.19) 0.408
T3 (≥ 94.1)	197/245	0.73 (0.55, 0.98) 0.034	0.67 (0.48, 0.93) 0.017	204/191	1.14 (0.85, 1.54) 0.380	0.85 (0.61, 1.19) 0.353
*Selenium Categories*						
T1–T2 (< 94.1)	424/376	*Ref.*	*Ref.*	430/443	*Ref.*	*Ref.*
T3 (≥ 94.1)	197/245	0.69 (0.54, 0.89) 0.003	0.63 (0.48, 0.83) 0.001	204/191	1.12 (0.86, 1.44) 0.399	0.92 (0.69, 1.22) 0.563
**First ischemic stroke**						
*Selenium tertiles*						
T1 (< 79.1 ng/mL)	179/171	*Ref.*	*Ref.*	181/192	*Ref.*	*Ref.*
T2 (79.1 to < 94.1)	191/157	1.16 (0.85, 1.57) 0.343	1.17 (0.82, 1.66) 0.387	181/183	1.05 (0.79, 1.40) 0.716	0.90 (0.64, 1.25) 0.514
T3 (≥ 94.1)	168/210	0.74 (0.55, 1.01) 0.058	0.67 (0.47, 0.95) 0.027	179/166	1.17 (0.85, 1.60) 0.330	0.84 (0.58, 1.21) 0.347
*Selenium categories*						
T1–T2 (< 94.1)	370/328	*Ref.*	*Ref.*	362/375	*Ref.*	*Ref.*
T3 (≥ 94.1)	168/210	0.69 (0.53, 0.90) 0.006	0.61 (0.45, 0.83) 0.001	179/166	1.14 (0.86, 1.50) 0.362	0.89 (0.65, 1.22) 0.479
**First hemorrhagic stroke**						
*Selenium tertiles*						
T1 (< 79.1 ng/mL)	26/22	*Ref.*	*Ref.*	32/32	*Ref.*	*Ref.*
T2 (79.1 to < 94.1)	26/26	0.81 (0.33, 2.00) 0.646	0.88 (0.25, 3.04) 0.840	34/35	0.98 (0.46, 2.12) 0.962	0.48 (0.17, 1.37) 0.172
T3 (≥ 94.1)	29/33	0.72 (0.32, 1.60) 0.420	0.80 (0.25, 2.58) 0.714	24/23	1.05 (0.43, 2.57) 0.919	0.58 (0.17, 1.92) 0.368
*Selenium categories*						
T1–T2 (< 94.1)	52/48	*Ref.*	*Ref.*	66/67	*Ref.*	*Ref.*
T3 (≥ 94.1)	29/33	0.80 (0.41, 1.54) 0.506	0.86 (0.33, 2.26) 0.761	24/23	1.06 (0.54, 2.10) 0.862	0.95 (0.36, 2.46) 0.910

*Abbreviations*: *T* tertile, *OR* odds ratio, *CI* confidence interval

^a^Model 1 is conditioned on the matching factors of age and sex; model 2 is conditioned on the matching factors of age and sex, as well as adjusted for BMI, baseline SBP, baseline DBP, smoking status, alcohol consumption, labor intensity, baseline total homocysteine, vitamin E, fasting glucose, estimated glomerular filtration rate (eGFR), anti-platelet drugs, lipoprotein-lowering drugs, glucose-lowering drugs, anti-hypertensive drugs, self-reported hypertension, self-reported diabetes, self-reported atrial fibrillation, and self-reported hyperlipidemia

^b^ Adjusted *P*-interaction between sex and plasma selenium (T3, ≥ 94.1 ng/mL *vs.* T1–2, < 94.1 ng/mL) on first total stroke = 0.029

Similar effects of sex on the Se-first ischemic stroke association were also observed and are displayed in Table 4. However, no significant association was found between plasma Se and first hemorrhagic stroke risk among either males or females (Table 4).

### Stratified analysis by potential effect modifiers in male and female participants

Stratified analyses were conducted to explore potential modifiers affecting the association between plasma Se (tertile 3, ≥ 94.1 *vs.* tertile 1–2, < 94.1 ng/mL) and first total stroke risk among male participants (Table 5). A stronger non-linear negative association between baseline plasma Se and first total stroke was found among males with higher vitamin E levels than among those with lower vitamin E levels (≥ 13.5 μg/mL; OR 0.39; 95% CI 0.25, 0.60; *vs.* < 13.5 μg/mL; OR 0.85; 95% CI 0.62, 1.17; *P* for interaction = 0.007). None of the other variables, including age (< 70 *vs.* ≥ 70 years), BMI (< 24 *vs.* ≥ 24 kg/m^2^), current smoking (yes *vs.* no), current alcohol consumption (yes *vs.* no), baseline SBP (< 140 *vs.* ≥ 140 mmHg), fasting glucose (< 6.1 *vs.* ≥ 6.1 mmol/L or diabetes), TC (< 5.8 [median] *vs.* ≥ 5.8 mmol/L), TG (< 1.2 [median] *vs.* ≥ 1.2 mmol/L), eGFR (< 90 *vs.* ≥ 90 mL/min/1.73 m^2^), and total homocysteine (< 12.5 [median] *vs.* ≥ 12.5 μmol/L), were found to modify the association between plasma Se (tertile 3, ≥ 94.1 *vs.* tertile 1–2, < 94.1 ng/mL) and the risk of first stroke in males (*P* for all interactions > 0.05).

**Table 5:** Stratified analysis of the association between plasma selenium concentrations (T3, ≥ 94.1 ng/mL *vs.* T1–2, < 94.1 ng/mL) and incident risk of first total stroke in males

Subgroups	No. of cases/no. of controls		^a^Adjusted model	*P* for interaction
	Selenium ≥ 94.1 ng/mL	Selenium < 94.1 ng/mL	OR (95% CI)	
Age, years				0.288
< 70	98/110	173/161	0.73 (0.50, 1.08)	
≥ 70	99/135	251/215	0.54 (0.38, 0.76)	
Body mass index, kg/m^2^				0.067
< 24	44/89	151/152	0.39 (0.24, 0.63)	
≥ 24	153/156	273/224	0.75 (0.55, 1.02)	
Current smoking				0.739
No	95/136	233/231	0.72 (0.51, 1.01)	
Yes	102/109	191/145	0.54 (0.36, 0.80)	
Current alcohol drinking				0.230
No	98/106	232/207	0.74 (0.51, 1.06)	
Yes	99/139	192/169	0.53 (0.37, 0.77)	
SBP, mmHg				0.759
< 140	46/92	124/156	0.60 (0.38, 0.96)	
≥ 140	151/153	300/220	0.64 (0.47, 0.88)	
Glucose, mmol/L				0.898
< 6.1	108/168	274/284	0.66 (0.48, 0.90)	
≥ 6.1 or diabetes^b^	89/77	150/92	0.66 (0.42, 1.02)	
TC, mmol/L				0.402
< 5.8	95/107	262/236	0.77 (0.54, 1.10)	
≥ 5.8	102/138	162/140	0.53 (0.36, 0.78)	
TG, mmol/L				0.611
< 1.2	115/160	246/255	0.71 (0.52, 0.98)	
≥ 1.2	82/85	178/121	0.55 (0.36, 0.84)	
eGFR, mL/min/1.73 m^2^				0.753
< 90	64/68	173/141	0.71 (0.45, 1.10)	
≥ 90	133/177	251/235	0.61 (0.45, 0.83)	
tHcy, μmol/L				0.117
< 12.5	89/105	129/142	0.81 (0.54, 1.22)	
≥ 12.5	108/140	293/234	0.55 (0.39, 0.76)	
Vitamin E, μg/mL				0.007
< 13.5	117/132	270/277	0.85 (0.62, 1.17)	
≥ 13.5	80/113	154/99	0.39 (0.25, 0.60)	

*Abbreviations*: *TC*, total cholesterol, *T* tertile, *OR* odds ratio, *CI* confidence interval

^a^ORs of first total stroke in relation to serum selenium levels were calculated using multivariate logistic regression models. Each subgroup analysis was adjusted, if not stratified, for age, BMI, baseline SBP, baseline DBP, smoking status, alcohol consumption, labor intensity, baseline total homocysteine, vitamin E, fasting glucose, estimated glomerular filtration rate (eGFR), anti-platelet drugs, lipoprotein-lowering drugs, glucose-lowering drugs, anti-hypertensive drugs, self-reported hypertension, self-reported diabetes, self-reported atrial fibrillation, and self-reported hyperlipidemia

^b^Diabetes was defined as a self-reported history of diabetes mellitus, use of anti-diabetic medications, or fasting glucose ≥ 7.0 mmol/L

Furthermore, none of the above variables significantly modified the association of plasma Se and the risk of first total stroke in female participants (*P* for all interactions > 0.05) (Supplemental Table 3).

## Discussion

This nested case-control study demonstrates that higher baseline plasma selenium (Se) is associated with lower risks of first total stroke and first ischemic stroke in males but not in females. Plasma vitamin E levels significantly modified the association between plasma Se and first total stroke in males. Furthermore, no significant association was found between plasma Se and first hemorrhagic stroke risk in either male or female participants.

Conflicting findings of the association between plasma Se and the risk of stroke have been reported by previous studies. A nested case-control study [38] enrolling 1304 stroke cases with a median plasma Se concentration of 64.9 ng/mL found that higher plasma Se levels were significantly associated with a lower risk of hemorrhagic stroke, but not ischemic stroke; the odds ratios (ORs) of hemorrhagic and ischemic stroke were 0.68 (95% CI 0.51, 0.91) and 0.92 (95% CI 0.82, 1.05) in the higher Se level tertile 3 (compared with tertile 1). One case-control study [35] including 1277 ischemic stroke patients with a median plasma Se concentration of 81.1 ng/mL indicated that higher plasma Se levels were associated with a decreased risk of ischemic stroke, where the OR for those with higher Se levels in quartile 4 (compared with quartile 1) was 0.10 (95% CI 0.06, 0.17). Moreover, the Canadian Health Measures Survey (CHMS 2007–2011) enrolling 7065 adult subjects with a median whole blood Se concentration of 184 ng/mL and the National Health and Nutrition Examination Study (NHANES 2011–2012) enrolling 5030 adult subjects with a median whole blood Se concentration of 181 ng/mL found inverse, cross-sectional associations between whole blood Se and the prevalence of stroke, and the Inuit Health Survey (IHS) enrolling 49 stroke cases with a median whole blood Se concentration of 260 ng/mL indicated a reverse relation of whole blood and dietary Se levels with stroke but revealed an L-shaped relationship [33, 34]. However, the Reasons for Geographic and Racial Differences in Stroke Study (REGARDS) [36] revealed that higher environmental Se levels were associated with increased stroke risk; the hazard ratio (HR) for those in quartile 4 (0.45–2.20 ppm) of environmental Se exposure (compared with quartile 1, 0.10–0.30 ppm) was 1.33 (95% CI 1.09, 1.62). It is noteworthy that all of these studies used different sources (plasma, whole blood, diet, and environment) of Se levels to assess the association between Se levels and stroke, which might be one reason for the discrepancy in these findings. Different Se statuses at baseline might be another important reason for the discrepancy in these findings.

Several studies have also explored the association between Se and stroke mortality specifically. A cohort study [37] enrolling 23 stroke death cases among 1100 Finnish males with a mean plasma Se concentration of 55.4 ng/mL found that low Se selenium (< 45 μg/L) was associated with a higher risk of stroke mortality, reporting an adjusted relative risk of 3.7 (95% CI 1.0, 13.1). The NHANES III cohort study [41] including 13,887 participants with a mean plasma Se concentration of 125.6 ng/mL found that the association curve for Se and stroke mortality had a reversed U-shape. However, another cohort study including 1103 Chinese participants with a mean plasma Se concentration of 73 ng/mL found no significant association of plasma Se levels and stroke mortality [42]. Notably, these studies focused on stroke mortality, and these findings might not represent the association of plasma Se levels and first stroke risk. Similarly, prospective associations between Se status/intake and cardiovascular outcomes remain inconclusive [43–45]. The Selenium and Vitamin E Cancer Prevention Trial (SELECT) with a median plasma Se concentration of 136 ng/mL at baseline and the Nutritional Prevention of Cancer Trial (NPC) with a median plasma Se concentration of 115 ng/mL at baseline found no beneficial effects of Se supplementation on cardiovascular outcomes in relatively similar North American populations. However, an interaction of Se status and cancer risk could be found in NPC but not in SELECT. A previous meta-analysis enrolling 16 prospective studies indicated that the Se level was negatively related to cardiovascular risk within a narrow Se range of 55–145 ng/mL [46]. Therefore, the findings of the studies mentioned above suggested that baseline Se status was an important factor affecting the association between Se and human health. The median serum Se level in the present study was 86.7 ng/mL, which was slightly higher than those in European populations (German, mean Se level of 73.9 ng/mL; Sweden, mean serum Se level of 67.1 ng/mL) but significantly lower than those in the American population (mean serum Se level of 137.1 ng/mL). Several studies found an inverse relationship between the Se level and cardiovascular disease among some Chinese and European populations with low Se levels, which was not observed in other studies performed among some American populations with high Se levels [35, 37, 47]. Thus, the present study found a negative association between serum Se and stroke risk at a relatively low level of Se at baseline, which further suggested that baseline Se status should be taken into account when evaluating the association between Se and stroke risk. None of the previous studies reported a sex difference in the association between plasma Se and stroke risk, and the results of these studies remain inconclusive. The present study provides an opportunity to explore the possible relationship between plasma Se and first stroke and to examine the potential effect modifiers in a community-based Chinese population.

Our current study provides three new insights into the field. First, to the best of our knowledge, this is the first study to find a significant non-linear, inverse association between plasma Se and first total stroke and first ischemic stroke risks in males but not in females. The differences in the primary outcome (first stroke) between the sexes in our study may be explained by the differences in the way Se is metabolized between the male and female reproductive systems. The retention rate for Se is highly efficient in the testes, while it appears that the female reproductive system does not retain significant levels of Se as efficiently [48–50]. The interaction of Se with the thyroid axis may be another reason for the differences. Wang et al. [25] proved strong sex-specific differences in the risk and development of hyperthyroidism in relation to baseline Se intake. Se deficiency might constitute a risk factor for hyperthyroidism in males, but no substantial association was found between hyperthyroidism prevalence and Se status in females. Hyperthyroidism has been reported to be associated with a 2- to 3-fold increased risk of ischemic and non-ischemic stroke [51]. Therefore, we speculate that high Se levels may reduce the adverse effects in males due to Se deficiency, which may explain why the non-linear inverse association between serum Se and first stroke was mainly found among males. Further prospective studies are needed to verify this differential association by sex.

Second, we observed a sharp decline in the risk of first stroke when plasma Se was over 94.1 ng/mL, suggesting that this value might serve as a high plasma Se cut-off point marking a decreased risk of first stroke or a low plasma Se cut-off point marking an increased risk of first stroke. This cut-off value agrees with a previous study that reported that plasma Se > 90 μg/L was sufficient to optimize the functions of selenoproteins [52], which are believed to carry out the functions of Se in its role of Se compounds. Schomburg Lutz et al. [53] also reported that deficiency of selenoprotein P, the main carrier of Se to target organs and reduces tissue oxidative stress both directly and by delivering Se to protective selenoproteins, was associated with an increased risk of stroke in a North European population without a history of cardiovascular disease. Se concentrations approximately 110–125 ng/mL and 90–100 ng/mL are needed to maximize the expression of selenoprotein P and GPX3, respectively, both of which are biomarkers for a replete Se status [54, 55]. From this perspective, it is reasonable that no significant inverse relationship between serum selenium level and stroke risk was observed in the American populations with baseline selenium above 130 ng/mL because the Se level is replete for the populations, whereas a significant negative association between Se level and stroke risk was found in the present study with baseline selenium of approximately 86.66 ng/mL because the Se level is inadequate for this population. The reference value of serum Se concentration used in the present study was 50–120 ng/mL, which was based on the evaluation of data from the literature summarized by the Human Biomonitoring Commission, 2002 [56]. However, it should also be noted that the cut-off value of Se in the present study is still within the normal range for human plasma Se (50–120 ng/mL) [39], and our findings were found mainly among a population with normal Se levels, with a prevalence of plasma Se < 50, 50 to 120, and > 120 ng/mL of 1.2%, 95.1%, and 3.7% in this study (Supplemental Table 1). Therefore, the use of the cut-off value and normal range for human plasma Se (50–120 ng/mL) among stroke patients needs careful consideration. Our results, if further confirmed, might have vital clinical and public health implications for community residents in China.

Third, our study is the first to indicate a stronger non-linear negative relationship between plasma Se and first stroke in male participants with higher plasma vitamin E levels (≥ 13.5 μg/mL) than in those with lower plasma vitamin E levels (<13.5 μg/mL). This finding suggests that higher plasma Se and higher plasma vitamin E levels may jointly decrease the first stroke risk. A previous meta-analysis demonstrated a significant inverse association between dietary vitamin E intake and stroke risk, where a higher dietary vitamin E intake was associated with a lower risk of stroke [57]. The exact mechanisms underlying a high Se × high vitamin E interaction remain unclear. One plausible biological explanation for the interaction may be that both Se and vitamin E belong to the vital antioxidants and participate in protecting against brain oxidative stress [58], one of the hallmarks of stroke. Accordingly, high plasma vitamin E and Se levels may share some cellular and molecular mechanisms with the pathogenesis of stroke, which could cause the interaction in the non-linear negative relationship between plasma Se and first stroke in males. Further studies are warranted to verify this hypothesis.

While the mechanisms underlying the effect of Se on first stroke remain inconclusive, an association seems reasonable due to several vital biological functions of Se. Hosnedlova et al. [59] demonstrated that Se mainly exerts a protective effect against oxidative lipid damage in the brain and modulates neurotoxicity and oxidative stress in nervous tissue. Furthermore, modulation of inflammatory and metabolic signaling, as well as preservation of mitochondrial function, may also be involved in the protective role of Se in stroke [60, 61]. Se deficiency in heart failure patients was independently associated with impaired exercise tolerance and a 50% higher mortality rate and impaired mitochondrial function in vitro in human cardiomyocytes [62]. Ishrap et al. [63] reported that pharmacological Se supplementation might have an unexpected ability to drive adaptive transcription to counter ferroptosis and protect neurons after stroke both in vitro and in vivo in animal models. Further studies are needed to illuminate the mechanisms underlying the association between plasma Se and stroke.

Several possible limitations in this study should be mentioned. First, the plasma Se concentrations only represent the baseline Se levels of all participants; more frequent measurements during the follow-up would have strengthened the accuracy of our results. Second, only plasma Se concentrations were used as the biomarker of Se levels in our study; other biomarkers, including whole blood and urinary Se concentrations, should also be considered when performing a sensitivity analysis to confirm our findings. Third, this was a nested, case-control study, not a cohort study, with a relatively small sample size from a community-based population, and all stratified analyses were not prespecified; thus, this work was a product of hypothesis generation. Although the use of first stroke in this study may be reasonable to avoid reverse causation, it cannot entirely clarify the prospective relationship between Se and first stroke, and further larger-scale cohort studies are needed to verify this issue. Fourth, the findings were observed among subjects with H-type hypertension, which might not be entirely representative of the general population; thus, the findings of this study might not be applied to other populations. Finally, since Se is renally eliminated under the influence of diuretics, we adjusted for all anti-hypertensive drugs together and did not analyze the effects of diuretics separately on the association; thus, further analysis is needed to clarify this issue.

In summary, we found a significant non-linear, inverse association between baseline plasma Se and the risks of first stroke and first ischemic stroke in males but not in females. Plasma vitamin E level was a potential modifier affecting this association. In addition, no significant association between plasma Se and first hemorrhagic stroke was found among either sex.

### Perspectives and significance

Data from the present study show that there was a sex difference in the association between plasma Se concentration and first stroke risk. This relationship existed among males but not in females. Thus, our findings suggest that we should take sex differences into account and stratify the data by sex when evaluating the association between plasma Se and first stroke risk in the future. If further confirmed, our findings may provide important data for clinical and nutritional guidelines on the primary prevention of first stroke among males by taking plasma Se into account to serve as a potentially modifiable risk factor and a possible biomarker for the purposes of monitoring and intervention. Furthermore, one future direction of this work is to clarify the potential mechanisms of sex disparities in plasma Se and first stroke risk.

## Supplementary Information

**Additional file 1: Supplemental Table 1.** Distributions of plasma selenium concentrations. **Supplemental Table 2.** Characteristics of study participants by tertiles of baseline plasma selenium concentrations^a^. **Supplemental Table 3.** Stratified analysis of the association between plasma selenium concentrations (T3, ≥94.1 ng/mL *vs.* T1-2, <94.1 ng/mL) and incident risk of first total stroke in females. **Supplemental Figure 1.** Flow chart of the study participants using a nested case-control design. *1401 controls were individually matched with 1401 cases by age (within 1 year), sex and village at a 1:1 ratio. Abbreviations: CHHRS: China H-type Hypertension Registry Study. **Supplemental Figure 2.** Distributions of plasma selenium (A) and vitamin E (B) levels by sex. **Supplemental Figure 3.** The association between baseline plasma selenium and the risk of first hemorrhagic stroke. Odds ratios for first hemorrhagic stroke in the (A) total population, (B) males, and (C) females by plasma selenium levels. In addition to the matching factors (age and sex), the splines also adjusted for BMI, baseline SBP, baseline DBP, smoking status, alcohol consumption, labor intensity, baseline total homocysteine, vitamin E, fasting glucose, estimated glomerular filtration rate (eGFR), anti-platelet drugs, lipoprotein-lowering drugs, glucose-lowering drugs, anti-hypertensive drugs, self-reported hypertension, self-reported diabetes, self-reported atrial fibrillation, and self-reported hyperlipidemia.

## Data Availability

All data are available from the corresponding author upon request.

## Abbreviations

CHHRS: China H-type Hypertension Registry Study
CDC: Chinese Centers for Disease Control and Prevention
OR: Odds ratio
CI: Confidence intervals
BMI: Body mass index
SBP: Systolic blood pressure
DBP: Diastolic blood pressure
tHcy: Total homocysteine
eGFR: Estimated glomerular filtration rate
TC: Total cholesterol
TG: Triglycerides
HDL-C: High-density lipoprotein-cholesterol
