# Supplementary material for: Sex difference in the association between plasma selenium and first stroke: a community-based nested case-control study
Source: Biol Sex Differ. 2021 May 29;12:39. doi: 10.1186/s13293-021-00383-2 (PMC8164764; doi:10.1186/s13293-021-00383-2)
Supplement: Supplementary file 1 — Additional file 1: Supplemental Table 1. Distributions of plasma selenium concentrations. Supplemental Table 2. Characteristics of study participants by tertiles of baseline plasma selenium concentrationsa. Supplemental Table 3. Stratified analysis of the association between plasma selenium concentrations (T3, ≥94.1 ng/mL vs. T1-2, <94.1 ng/mL) and incident risk of first total stroke in females. Supplemental Figure 1. Flow chart of the study participants using a nested case-control design. *1401 controls were individually matched with 1401 cases by age (within 1 year), sex and village at a 1:1 ratio. Abbreviations: CHHRS: China H-type Hypertension Registry Study. Supplemental Figure 2. Distributions of plasma selenium (A) and vitamin E (B) levels by sex. Supplemental Figure 3. The association between baseline plasma selenium and the risk of first hemorrhagic stroke. Odds ratios for first hemorrhagic stroke in the (A) total population, (B) males, and (C) females by plasma selenium levels. In addition to the matching factors (age and sex), the splines also adjusted for BMI, baseline SBP, baseline DBP, smoking status, alcohol consumption, labor intensity, baseline total homocysteine, vitamin E, fasting glucose, estimated glomerular filtration rate (eGFR), anti-platelet drugs, lipoprotein-lowering drugs, glucose-lowering drugs, anti-hypertensive drugs, self-reported hypertension, self-reported diabetes, self-reported atrial fibrillation, and self-reported hyperlipidemia. [file 13293_2021_383_MOESM1_ESM.doc]

**Supplemental Materials**

**Supplemental Table 1. Distributions of plasma selenium concentrations.**

| Group | N | Distributions | | | | | | Deficiency^a^, N (%) | | |
| --- | --- | --- | --- | --- | --- | --- | --- | --- | --- | --- |
|  |  | Mean±SD | Minimum | 25% | Median | 75% | Maximum | Deficiency | Normal | Excess |
| Total | 2510 | 87.2 ± 18.6 | 37.7 | 74.9 | 86.7 | 97.8 | 279.9 | 31 (1.2) | 2386 (95.1) | 93 (3.7) |
| Male | 1242 | 87.7 ± 18.6 | 38.8 | 75.4 | 87.1 | 98.5 | 279.9 | 13 (1.0) | 1182 (95.2) | 47 (3.8) |
| Female | 1268 | 86.8 ± 18.6 | 37.7 | 74.5 | 86.1 | 96.9 | 216.2 | 18 (1.4) | 1204 (95.0) | 46 (3.6) |

^a^ According to the definitions of reference value for plasma selenium by previous studies, the cut-off value (50-120 ng/mL) of plasma selenium concentrations was used to stratify our participants.

**Supplemental Table 2. Characteristics of study participants by tertiles of baseline plasma selenium concentrations.a**

| Characteristics | Selenium, ng/mL | | | *P* value |
| --- | --- | --- | --- | --- |
|  | T1 (<79.1) | T2 (79.1 to <94.1) | T3 (≥94.1) |  |
| Age, y | 71.3 ± 8.2 | 70.3 ± 8.3 | 70.7 ± 7.7 | 0.025 |
| Male, n (%) | 399 (47.7) | 401 (48.0) | 442 (52.8) | 0.062 |
| Body mass index, kg/m2 | 25.8 ± 4.4 | 26.1 ± 3.9 | 26.7 ± 4.0 | <0.001 |
| Current smoking, n (%) | 166 (19.8) | 173 (20.7) | 212 (25.3) | 0.014 |
| Current alcohol drinking, n (%) | 173 (20.7) | 198 (23.7) | 241 (28.8) | <0.001 |
| Baseline SBP, mmHg | 152.8 ± 23.9 | 153.0 ± 23.3 | 153.9 ± 22.0 | 0.584 |
| Baseline DBP, mmHg | 84.6 ± 12.9 | 85.59 ± 12.1 | 85.62 ± 11.9 | 0.127 |
| Self-reported hypertension, n (%) | 421 (50.3) | 445 (53.2) | 446 (53.3) | 0.376 |
| Self-reported diabetes, n (%) | 110 (13.1) | 132 (15.8) | 182 (21.7) | <0.001 |
| Self-reported hyperlipidemia, n (%) | 76 (9.1) | 90 (10.8) | 98 (11.7) | 0.207 |
| Self-reported atrial fibrillation, n (%) | 10 (1.2) | 14 (1.7) | 22 (2.6) | 0.084 |
| Hypertension, n (%)^b^ | 657 (78.5) | 672 (80.4) | 687 (82.1) | 0.182 |
| Labor intensity, n (%) |  |  |  | <0.001 |
| Mild | 591 (70.6) | 622 (74.4) | 672 (80.3) |  |
| Moderate | 183 (21.9) | 174 (20.8) | 131 (15.7) |  |
| Severe | 63 (7.5) | 40 (4.8) | 34 (4.1) |  |
| **Medication use, n (%)** | | | | |
| Antiplatelet drugs | 30 (3.6) | 25 (3.0) | 28 (3.4) | 0.792 |
| Lipid-lowering drugs | 9 (1.1) | 20 (2.4) | 15 (1.8) | 0.121 |
| Glucose-lowering drugs | 78 (9.3) | 102 (12.2) | 121 (14.5) | 0.005 |
| Antihypertensive drugs | 368 (44.0) | 392 (46.9) | 392 (46.8) | 0.390 |
| **Laboratory results** | | | | |
| TC, mmol/L | 5.6 ± 1.2 | 5.8 ± 1.2 | 6.1 ± 1.2 | <0.001 |
| TG, mmol/L | 1.4 ± 0.8 | 1.4 ± 0.8 | 1.5 ± 0.9 | 0.086 |
| HDL-C, mmol/L | 1.6 ± 0.4 | 1.6 ± 0.4 | 1.7 ± 0.4 | <0.001 |
| Glucose, mmol/L | 5.9 ± 1.9 | 6.3 ± 2.4 | 6.6 ± 2.6 | <0.001 |
| Total homocysteine, μmol/L | 14.5 ± 8.0 | 13.6 ± 7.7 | 13.6 ± 5.5 | 0.011 |
| eGFR, mL/min/1.73 m^2^ | 91.4 ± 15.0 | 92.9 ± 13.9 | 93.2 ± 14.3 | 0.020 |
| Vitamin E, μg/mL | 13.5 ± 3.6 | 14.0 ± 4.0 | 14.7 ± 4.2 | <0.001 |
| Selenium, ng/mL | 68.4 ± 8.2 | 86.4 ± 4.2 | 106.9 ± 14.3 | <0.001 |

^a^ Variables are presented as the mean ± SD or n (%). **Abbreviations:** SBP, systolic blood pressure; DBP, diastolic blood pressure; TC, total cholesterol; TG, triglycerides; eGFR, estimated glomerular filtration rate; HDL-C, high-density lipoprotein-cholesterol. ^b^ Hypertension was defined as self-reported history of hypertension, use of antihypertensive drugs, or SBP ≥140 mmHg, or DBP ≥90 mmHg.

**Supplemental Table 3. Stratified analysis of the association between plasma selenium concentrations (T3, ≥94.1 ng/mL *vs.* T1-2, <94.1 ng/mL) and incident risk of first total stroke in females.**

| Subgroups | No. of cases / No. of controls | | ^a^Adjusted Model | *P* for interaction |
| --- | --- | --- | --- | --- |
|  | Selenium≥94.1 ng/mL | Selenium<94.1 ng/mL | OR (95% CI) |  |
| Age, y |  |  |  | 0.890 |
| <70 | 99/87 | 218/232 | 0.95 (0.65, 1.40) |  |
| ≥70 | 105/104 | 212/211 | 0.93 (0.65, 1.34) |  |
| Body mass index, kg/m^2^ |  |  |  | 0.819 |
| <24 | 33/40 | 100/123 | 0.87 (0.47, 1.61) |  |
| ≥24 | 171/151 | 330/320 | 0.96 (0.72, 1.29) |  |
| SBP, mmHg |  |  |  | 0.807 |
| <140 | 39/51 | 104/150 | 0.97 (0.57, 1.66) |  |
| ≥140 | 165/140 | 326/293 | 0.95 (0.71, 1.28) |  |
| Glucose, mmol/L |  |  |  | 0.205 |
| <6.1 | 89/99 | 253/313 | 1.11 (0.78, 1.58) |  |
| ≥6.1 or diabetes^b^ | 115/92 | 177/130 | 0.76 (0.51, 1.11) |  |
| TC, mmol/L |  |  |  | 0.410 |
| <5.8 | 77/61 | 207/209 | 1.10 (0.72, 1.68) |  |
| ≥5.8 | 127/130 | 223/234 | 0.86 (0.62, 1.20) |  |
| TG, mmol/L |  |  |  | 0.442 |
| <1.2 | 51/71 | 154/201 | 0.82 (0.52, 1.29) |  |
| ≥1.2 | 153/120 | 276/242 | 1.01 (0.73, 1.39) |  |
| eGFR, mL/min/1.73m^2^ |  |  |  | 0.743 |
| <90 | 68/55 | 157/138 | 1.12 (0.69, 1.81) |  |
| ≥90 | 136/136 | 273/305 | 0.89 (0.65, 1.22) |  |
| tHcy, μmol/L |  |  |  | 0.502 |
| <12.5 | 118/120 | 258/281 | 0.89 (0.63, 1.24) |  |
| ≥12.5 | 85/71 | 171/161 | 1.09 (0.72, 1.66) |  |
| Vitamin E, μg/mL |  |  |  | 0.355 |
| <13.5 | 56/58 | 170/172 | 0.88 (0.55, 1.42) |  |
| ≥13.5 | 148/133 | 260/271 | 1.02 (0.75, 1.39) |  |

^a^ ORs of first total stroke in relation to serum selenium levels were calculated using multivariate logistic regression models. Each subgroup analysis was adjusted, if not stratified, for age, BMI, baseline SBP, baseline DBP, smoking status, alcohol consumption, labor intensity, baseline total homocysteine, vitamin E, fasting glucose, estimated glomerular filtration rate (eGFR), antiplatelet drugs, lipoprotein-lowering drugs, glucose-lowering drugs, antihypertensive drugs, self-reported hypertension, self-reported diabetes, self-reported atrial fibrillation, and self-reported hyperlipidemia. ^b^ Diabetes was defined as self-reported history of diabetes mellitus, use of anti-diabetic medications, or fasting glucose ≥7.0 mmol/L. **Abbreviations:** TC, total cholesterol; T, tertile; OR, odds ratio; CI, confidence interval.


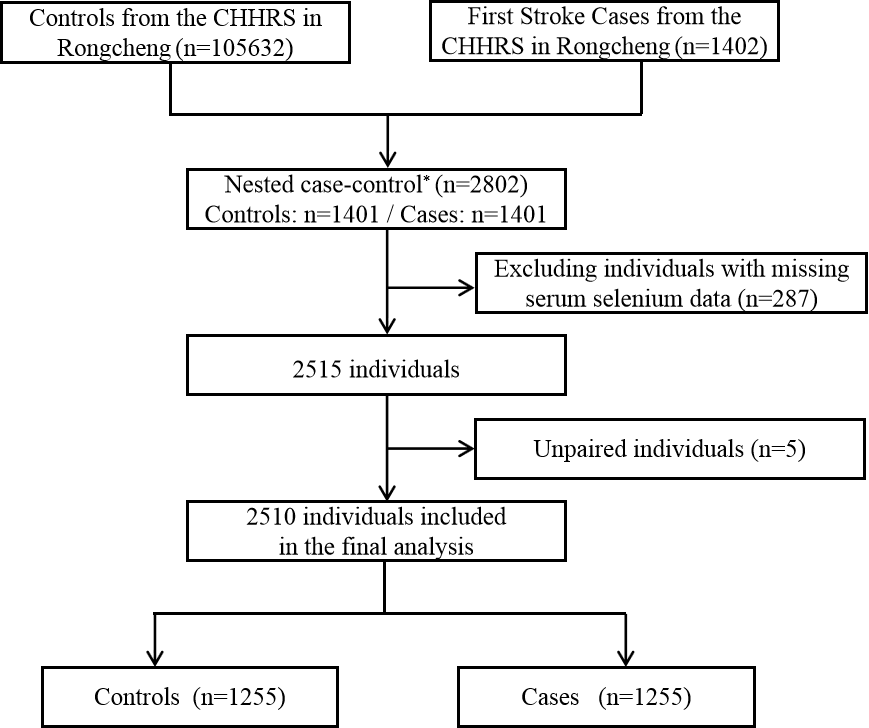


**Supplemental Figure 1**. Flow chart of the study participants using a nested case-control design.


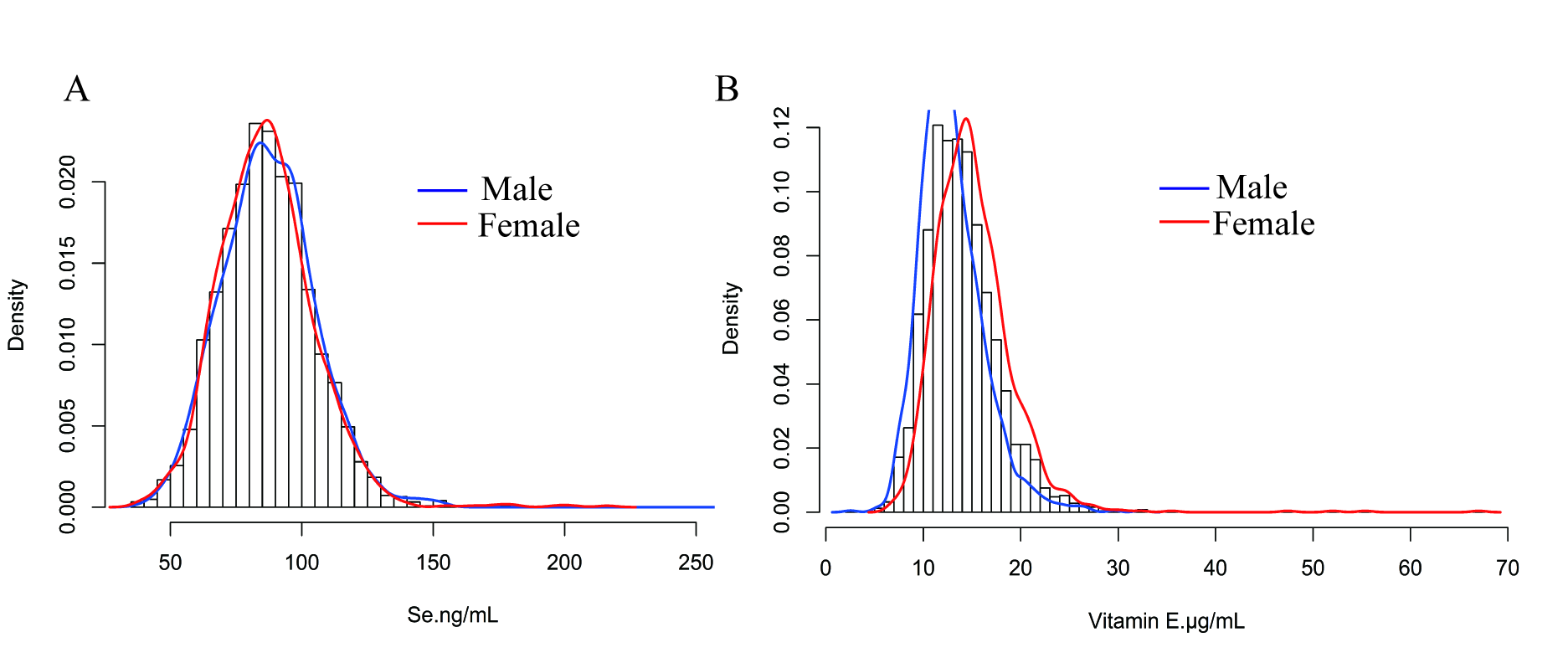
**Supplemental Figure 2. Distributions of plasma selenium (A) and vitamin E (B) levels by sex.**

**
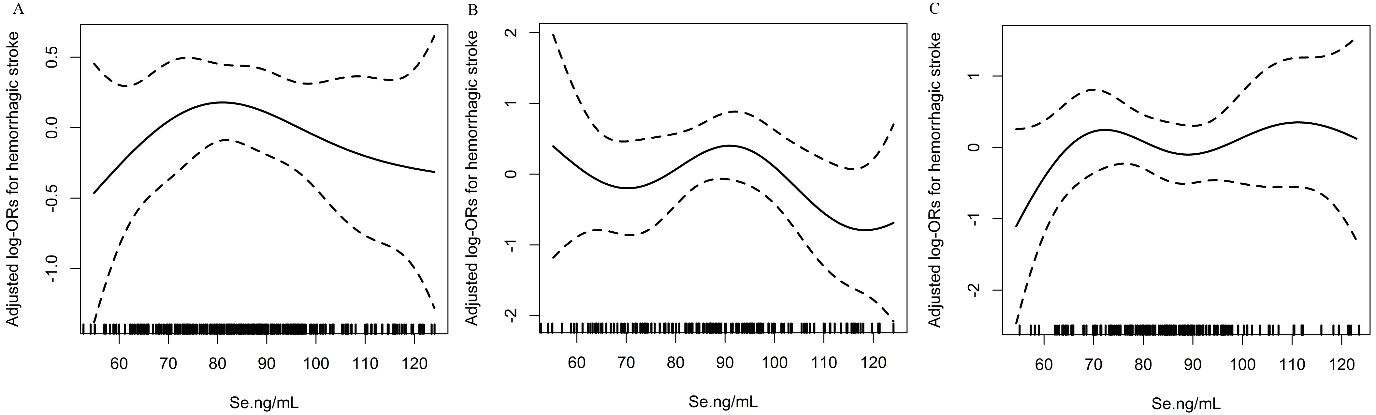
Supplemental Figure 3. The association between baseline plasma selenium and the risk of first hemorrhagic stroke.**
